# The complete mitochondrial genomes of two rice planthoppers, *Nilaparvata lugens* and *Laodelphax striatellus:* conserved genome rearrangement in Delphacidae and discovery of new characteristics of *atp8* and tRNA genes

**DOI:** 10.1186/1471-2164-14-417

**Published:** 2013-06-22

**Authors:** Kai-Jun Zhang, Wen-Chao Zhu, Xia Rong, Yan-Kai Zhang, Xiu-Lei Ding, Jing Liu, Da-Song Chen, Yu Du, Xiao-Yue Hong

**Affiliations:** 1 Department of Entomology, Nanjing Agricultural University, Nanjing 210095, China; 2 Beijing Genomics Institute, Shenzhen 518083, China

## Abstract

**Background:**

*Nilaparvata lugens* (the brown planthopper, BPH) and *Laodelphax striatellus* (the small brown planthopper, SBPH) are two of the most important pests of rice. Up to now, there was only one mitochondrial genome of rice planthopper has been sequenced and very few dependable information of mitochondria could be used for research on population genetics, phylogeographics and phylogenetic evolution of these pests. To get more valuable information from the mitochondria, we sequenced the complete mitochondrial genomes of BPH and SBPH. These two planthoppers were infected with two different functional *Wolbachia* (intracellular endosymbiont) strains (*w*Lug and *w*Stri). Since both mitochondria and *Wolbachia* are transmitted by cytoplasmic inheritance and it was difficult to separate them when purified the *Wolbachia* particles, concomitantly sequencing the genome of *Wolbachia* using next generation sequencing method, we also got nearly complete mitochondrial genome sequences of these two rice planthoppers. After gap closing, we present high quality and reliable complete mitochondrial genomes of these two planthoppers.

**Results:**

The mitogenomes of *N*. *lugens* (BPH) and *L*. *striatellus* (SBPH) are 17, 619 bp and 16, 431 bp long with A + T contents of 76.95% and 77.17%, respectively. Both species have typical circular mitochondrial genomes that encode the complete set of 37 genes which are usually found in metazoans. However, the BPH mitogenome also possesses two additional copies of the *trnC* gene. In both mitochondrial genomes, the lengths of the *atp8* gene were conspicuously shorter than that of all other known insect mitochondrial genomes (99 bp for BPH, 102 bp for SBPH). That two rearrangement regions (*trnC*-*trnW* and *nad6*-*trnP*-*trnT*) of mitochondrial genomes differing from other known insect were found in these two distantly related planthoppers revealed that the gene order of mitochondria might be conservative in Delphacidae. The large non-coding fragment (the A+T-rich region) putatively corresponding responsible for the control of replication and transcription of mitochondria contained a variable number of tandem repeats (VNTRs) block in different natural individuals of these two planthoppers. Comparison with a previously sequenced individual of SBPH revealed that the mitochondrial genetic variation within a species exists not only in the sequence and secondary structure of genes, but also in the gene order (the different location of *trnH* gene).

**Conclusion:**

The mitochondrial genome arrangement pattern found in planthoppers was involved in rearrangements of both tRNA genes and protein-coding genes (PCGs). Different species from different genera of Delphacidae possessing the same mitochondrial gene rearrangement suggests that gene rearrangements of mitochondrial genome probably occurred before the differentiation of this family. After comparatively analyzing the gene order of different species of Hemiptera, we propose that except for some specific taxonomical group (e.g. the whiteflies) the gene order might have diversified in family level of this order. The VNTRs detected in the control region might provide additional genetic markers for studying population genetics, individual difference and phylogeographics of planthoppers.

## Background

The mitochondrial genome is the most commonly used molecular marker for phylogenetic studies, population genetics and dynamics, phylogeography [1] and even for mitochondrial related insecticide/acaricide resistance [2]. It also can provide very favourable information at the genome level, such as the relative positions of different genes [3-7], strand asymmetry in nucleotide composition [8] and evolutionary patterns of the control region [9]. Furthermore, some special structural features such as minicircles could exist in some specified groups of insects [10,11].

The order Hemiptera is the largest group of the hemimetabolous insects [12] and includes three phylogenetically controversial suborders: Auchenorrhyncha, Sternorrhyncha, and Heteroptera [13]. The complete mitochondrial genome sequences have been determined for more than 300 species of insects so far. Within Hemiptera, although 47 complete or nearly complete mitochondrial genomes are available at NCBI (as of October 1, 2012), still very few species in some groups, especially in the suborder Auchenorrhyncha (7 species) and Sternorrhyncha (9 species) had been sequenced. In the Delphacidae, only one species has had its mitochondrial genome sequenced so far [14]. The phylogenetic relationships of this order at the superfamily and suborder levels are still controversial [14-18]. To solve these controversial phylogenetic problems in this order requires more information from mitochondrial genomes and even from nuclear genes [19].

The brown planthopper (BPH), *Nilaparvata lugens* and the small brown planthopper (SBPH), *Laodelphax striatellus* (Hemiptera: Delphacidae) are two of the most important pests in rice fields of many Asian countries. By transmitting virus diseases [20] and causing directly feeding damage, these two planthoppers cause serious yield losses of rice and economic loss for taking control measures each year [21]. These two planthoppers were also infected with the most widespread endosymbiont *Wolbachia*[22] which can manipulate the reproduction and affect the fitness of its host [23,24]. While sequencing the genomes of two different phenotype-inducing *Wolbachia* strains, we also obtained nearly the complete mitochondrial genomes of BPH and SBPH. The *Wolbachia* strain *w*Stri infecting SBPH can induce complete Cytoplasmic Incompatibility (CI), a reproductive modification that typically results in embryonic lethality between crosses of infected males and uninfected females. On the other hand, the *Wolbachia* strain *w*Lug infecting BPH cannot induce CI at all.

After gap-closing, the complete mitochondrial genomes of these two most important rice piercing and sucking type pests, BPH and SBPH were determined. We analyzed the nucleotide composition, codon usage and genomic rearrangement of these two mitochondrial genomes and compared them with mitochondrial genomes of other hemipteran species. Some special characteristics of mitochondria of these two planthoppers (for example, the variable number of tandem repeats (VNTRs) in ‘A + T’ rich regions; location of *trnH* gene) were verified using natural populations. In addition, comparing mitochondrial genome of SBPH sequenced in this study with a previously sequenced individual revealed a remarkable variability between these two individuals.

## Result and discussion

### Genome organization, structure and composition

**Figure 1:**
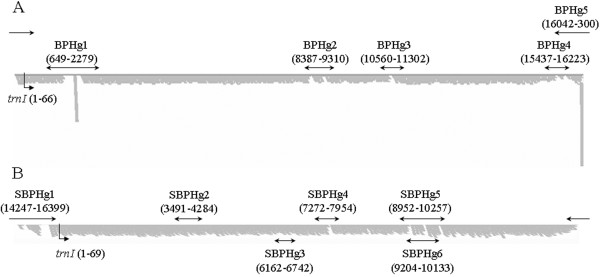
**Schematic map of reads assembly and gap closing of two mitochondrial genomes (A, *Nilaparvata lugens; *B, *Laodelphax striatellus*) in the present study.** The positions of *trnI* gene were shown. BPHg1 to BPHg5 and SBPHg1 to SBPHg6 indicated gaps/regions in the mitochondrial genomes of *N*. *Lugens* and *L*. *striatellus* respectively, and their locations were marked with the numbers in parentheses.

**Figure 2:**
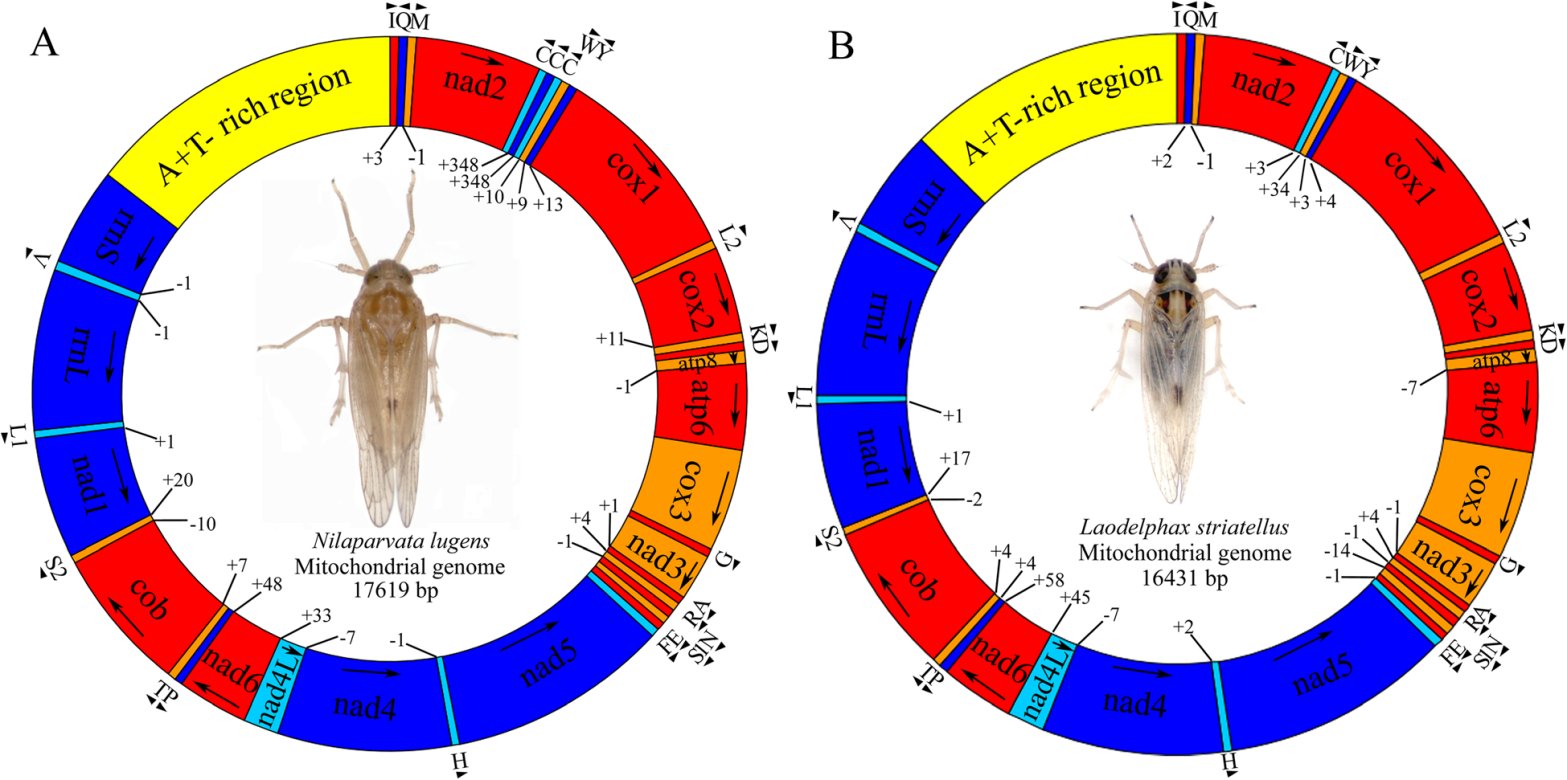
**Map of the mitochondrial genome of *Nilaparvata lugens *(A) and *Laodelphax striatellus *(B).** Genes coded in the J-strand (clockwise) are red or orange colored. Genes coded in the N-strand (counterclockwise) are blue or cyan colored. AT region (putative control region) is given in yellow colored. Gene names are the standard abbreviations used in this paper. Twenty two tRNA genes are indicated by the single letter IUPAC-IUB abbreviation for their corresponding amino acid in the draw. Arrows and arrowheads show the direction of the transcription of each gene. Numbers at gene junctions indicate the length of small non-coding regions where negative numbers indicate overlap between genes.

One scaffold containing 4 contigs of *N*. *lugens* (BPH) mtDNA and another scaffold containing 3 contigs of *L*. *striatellus* (SBPH) were identified in the assemblage of the *w*Lug and *w*Stri genome (Figure 1). After gap closing, the assembled result showed that the mitochondrial genomes of *N*. *lugens* and *L*. *striatellus* are typical circular DNA molecules with 17,619 bp and 16,431 bp in length, respectively. Both mitochondrial genomes include the entire set of 37 genes usually present in animal mtDNAs [1], i.e. 13 PCGs, 22 tRNA genes, and 2 ribosomal genes (Figure 2). Except for the whitefly *Trialeurodes vaporariorum*, the mitochondrial genome of BPH is the longest in Hemiptera (the longest in suborders Auchenorrhyncha and Heteroptera) which were reported so far. The unusual length of the mitochondrial genome in *N*. *lugens* is partly due to a long putative control region and a long repeat region containing three repeats of *trnC* gene (Figure 2, Additional file 1: Table S1). The repeat characteristic is supported by the depth of sequencing this region compared with other normal regions (Figure 1). However, it is unclear whether the gene duplication is correlated with function variation. Other species with duplicated genes in the mitochondrial genomes include *Leptotrombidium pallidum*[25] and *Metaseiulus occidentalis*[26].

All of the tRNA genes have the typical cloverleaf secondary structure, except for *trnS1* (AGN) of BPH, *trnS2* (UCN) of SBPH which harbor a simple loop in the dihydrouridine (DHU) arm and *trnH* of SBPH could have a loop-stem structure like *trnT* of BPH (Additional file 2: Figure S1, Additional file 3: Figure S2). The dihydrouridine (DHU) arm of *trnS1* (AGN) that forms a loop is found in many Hemiptera [17,18,27-30] and other metazoans [31]. For SBPH, the secondary structure in the dihydrouridine (DHU) arm in the *trnS1* (AGN) has a completed loop-stem structure while that of *trnS2* (UCN) has a loop. In contrast, the *trnS1* (AGN) has a loop and *trnS2* (UCN) has a completed loop-stem structure in previously reported SBPH [14]. Furthermore, it is similar that the *trnH* gene of SBPH which formed a weak loop-stem structure of the T-arm (TΨC) in this study possessed a stable loop-stem structural of the T-arm (TΨC) in previous study [14].

The length of all tRNA genes ranged from 56 bp for *trnS2* (UCN) (SBPH) and 57 bp for *trnS1* (AGN) (BPH) to 71 bp for *trnK* (BPH and SBPH) and *trnV* (BPH) (Additional file 1: Table S1 and Additional file 4: Table S2). As in other hemipteran species, BPH and SBPH harbor two rRNA genes, *rrnS* (748 bp for BPH and 747 bp for SBPH) and *rrnL* (1,219 bp for both). They are located between *trnL1* (CUN) and an A+T-rich region, separated by *trnV*.

### Mitochondrial gene order in Hemiptera

**Figure 3:**
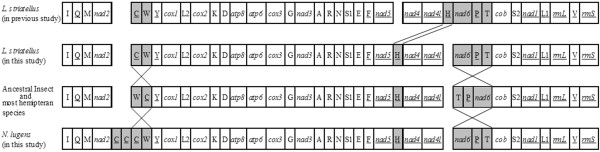
**Schematic representation of mitochondrial gene arrangements in *Nilaparvata lugens *and *Laodelphax striatellus*.** Underlined genes and tRNAs with letters below are encoded in the N-strand. Shaded boxes indicate genes involving mitochondrial genome arrangement. Most of the currently determined hemipteran species have the ancestral gene order. Previously sequenced *Laodelphax striatellus* has a translocation of *trnH* gene.

The mitochondrial gene orders of BPH and SBPH are significantly different from those of all other species of hemipteran insects. Two rearrangement regions (*trnC*-*trnW* and *nad6*-*trnP*-*trnT*) associated with tRNAs and PCGs were found in both of BPH and SBPH (Figure 3). The same gene order was found in the white-backed planthopper (WBPH), *Sogatella furcifera* (unpublished data). But the most striking feature was the different locations of the *trnH* gene in two individuals of SBPH. The *trnH* gene is located between *nad5* and *nad4* genes in this study just as in all other hemipteran species sequenced so far, but Song and Liang (2009) previously reported that this tRNA gene is located between *nad4L* and *nad6* genes (Figure 3) [14]. How could different gene orders appear in one species? Did the arrangement occur recently? We can not answer these questions yet.

However, the arrangement of these 37 genes, especially the arrangement of large genes that encode proteins and rRNAs, is usually conserved in the suborders Auchenorrhyncha and Heteroptera, except for the unique-headed bug *Stenopirates* sp. [28]. In this study, two long intergenic regions in upstream and downstream of *nad6* gene which contain a poly “T” and poly “A” stretch might provide some clues of rearrangement trace. In addition to rearrangement events involving large genes, rearrangement of *trnI* and *trnQ* in *Neuroctenus parus* and the rearrangement of *trnT* and *trnP* in *Physopelta gutta* and *Dysdercus cingulatus* have also been reported [32]. Except for these 4 species of Heteroptera, 2 planthoppers of Delphacidae in Auchenorrhyncha and 6 species of Aleyrodidae in the suborder Sternorrhyncha [33], the gene order of mitochondrial genomes of all the other species (27 species (22 families) in Heteroptera, 3 species (2 families) of Sternorrhyncha, 6 species (6 families) of Auchenorrhyncha) were found to be consistent with the mitochondrial gene order of the putative ancestral insect (Figure 3, Additional file 5: Table S3). Considering the fact that some families in the same superfamily have different gene arrangements of mitochondrial genome (Three families, Issidae, Fulgoridae and Flatidae of superfamily Fulgoroidea have the same gene order of mitochondria as the proposed ancestral insect, but members of the family Delphacidae have a different one), we proposed that excluding the whiteflies, the arrangement of genes in the mitochondria could be considered as a factor in inferring phylogenetic relationships of different species at the family level of hemipteran insects. In any case, further genome sequencing is necessary to establish whether this feature is a mitochondrial signature of the whole order.

### Protein-coding genes (PCGs) and codon usage patterns

The mtDNAs of BPH and SBPH contain the full set of PCGs usually presented in animal mtDNA. Except for the *nad6* gene, PCGs are arranged along the genome according to the proposed standard order of Insects [1] (Figure 2). All PCGs have typical start codons. Both of the putative start codons ATG and ATT are used in BPH and SBPH (Additional file 1: Table S1, Additional file 4: Table S2). Compared with SBPH, BPH has one more kind of initiation codon ATA in *nad3* and *atp6*, while ATA was not observed in SBPH. The *cox1*, *cox3*, *atp6*, and *nad5* genes of these two mtDNAs have incomplete stop codons. For the *cox2* gene, these two planthoppers have different stop codons, TAA for BPH and TAG for SBPH (Additional file 1: Table S1, Additional file 4: Table S2). The *atp8* and *atp6* of BPH and SBPH are the only PCGs having different overlapping nucleotides (1 bp for BPH, 7bp for SBPH). This feature is common in hemipteran insects [17], and is similar to all lepidopteran mitochondrial genomes known [34] and many other animal mtDNAs [1].

It is worth mentioning that the lengths of *atp8* gene of planthoppers (99 bp for BPH, 102 bp for SBPH) were the shortest in insects known so far. In fact, the longest *atp8* gene (228 bp) was found in *Bemisia tabaci* (Hemiptera: Aleyrodidae) [34]. In almost all other insects, the length ranged from 138 bp (Hemiptera: Psyllidae: *Pachypsylla venusta*) [33] to 183 bp (Lepidoptera: Lymantriidae: *Lymantria dispar*) [35]. In most insects, *atp8* gene had a length of about 160 bp. Taking into account the length of *atp8* gene of planthoppers discovered in this study, it seems that the length distribution of *atp8* gene in Hemiptera was wider (From 99 bp to 228 bp) than that of all other orders of insects reported so far. The sequence alignment of *atp8* gene with other insects revealed that nearly 60 bp length of sequence in the 3′end of this gene was lost in these two planthoppers. Does the short length of this gene change the structure of its encoded protein? Does length change of this gene alter the functions or efficiency of this gene? These aspects are worth further studying.

**Figure 4:**
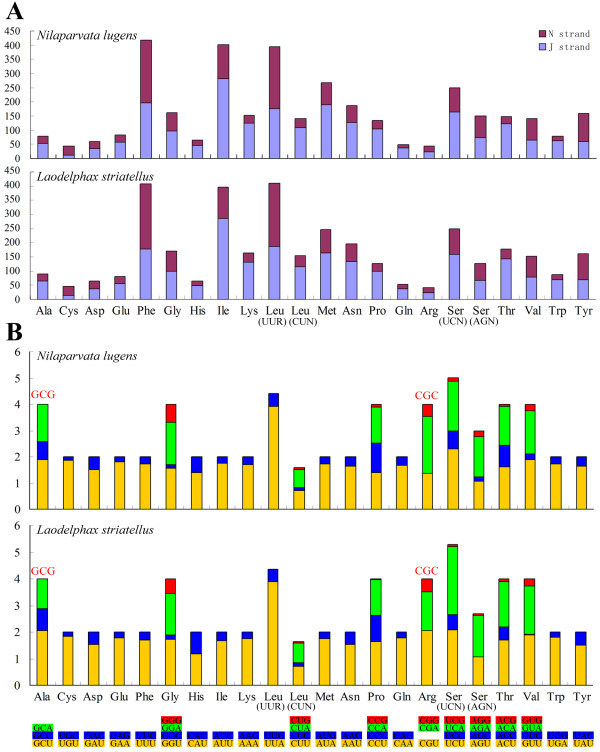
**Codon usage pattern (A) and the relative synonymous codon usage (RSCU) (B) of *Nilaparvata lugens *and *Laodelphax striatellus *mitochondrial genomes.** Numbers to the left refer to the total number of codons (**A**) and the RSCU value (**B**). Codon families are provided on the X-axis. Absent codons of the two mitogenomes are provided at the top of columns.

The abundance of codon families and Relative Synonymous Codon Usage (RSCU) [36] in PCGs in BPH and SBPH mtDNAs are summarized in Figure 4. The total numbers of non-stop codons (CDs) used by these two analyzed mtDNAs of planthoppers are very similar, 3,608 for BPH and 3,613 for SBPH. The codon families exhibit a very similar behavior among these two species. The most frequently used amino acid is Leu (BPH: 14.86%; SBPH: 15.42%), followed by Phe (BPH: 11.59%; SBPH: 11.13%), Ile (BPH: 11.12%; SBPH: 10.79%), Ser (BPH: 11.06%; SBPH: 10.27%) and Met (BPH: 7.43%; SBPH: 6.70%). The proportion of these five amino acids is more than 54%. Two codons (GCG, CGC) were not observed in the PCGs (Figure 4). CGC codons were also absent in the PCGs in many other hemipteran species [32].

### Control region of mitochondrial genome of BPH and SBPH

**Figure 5:**
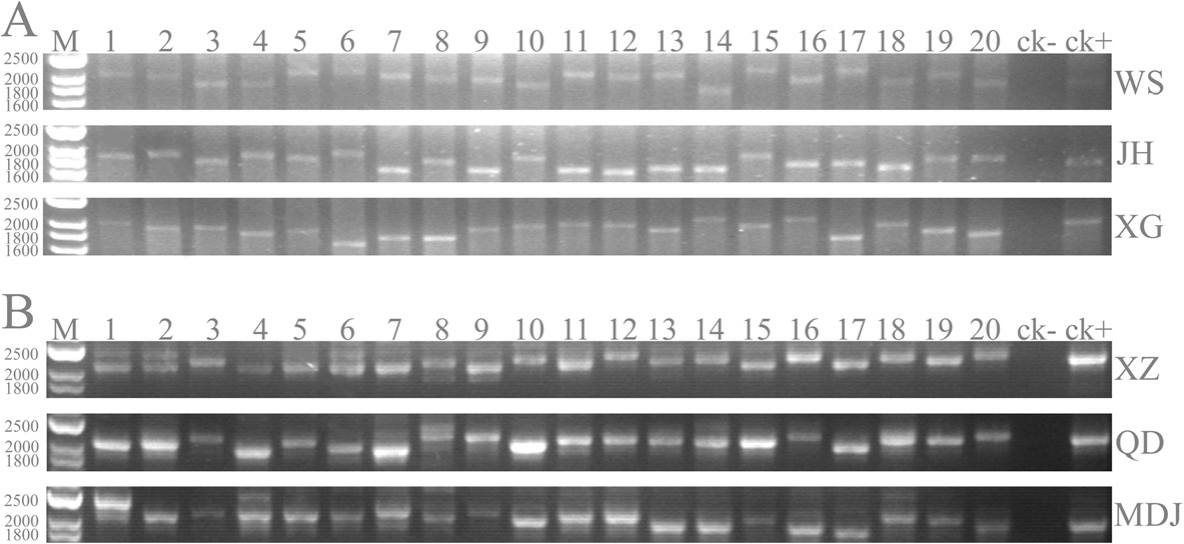
**PCR assays the fragment length polymorphisms of control region of *Nilaparvata lugens *(A) and *Laodelphax striatellus *(B).** M, molecular weight marker with sizes indicated on the left. The numbers above electropherogram indicated 20 individuals of each population were detected. The population codes (see Additional file 11: Table S7) are show on the right.

The control regions of BPH and SBPH mitogenomes are located at the conserved position between *rrnS* and *trnI*-*trnQ*-*trnM* gene cluster (Figure 2) with 79.3% and 83.2% A + T content, respectively. The lengths of control regions of mitochondrial genome of BPH and SBPH were obvious different (BPH: 2429 bp; SBPH: 2042 bp). The near 390 bp length difference was contributed by the different numbers of repeat unit. The repeat region of mitochondrial genome of BPH contained 55 times repeat of unit “GGAAAAAATGTCACGTTTTT(C/T)”, while similar repeat unit of SBPH “CACGATTTTTGGAAAAAATGT” showed 35 times repeat. But in repeat regions of both mitochondria contained incomplete repeat unit: the repeat 2 of BPH and the repeat 3, 14, 35 of SBPH were not complete. Within the same superfamily (Fulgoroidea) of these two planthoppers, the similar repeat units in control region had once reported in *Geisha distinctissima*[17] and *Sivaloka damnosus*[16], though the lengths of repeat regions were much shorter than that of SBPH and BPH. In contrast, a small number of long tandem repeats are commonly appeared in species of Heteroptera [18,28,32,37]. Actually, the variable number of tandem repeats (VNTRs) of mitochondrial genome of BPH and SBPH were discovered in natural populations (Figure 5). PCR amplification a fragment of mitochondrial genome containing the repeat regions of different individuals from three natural populations of each of these two planthopper species exhibited length polymorphism. The length polymorphism caused by variable repeat numbers was also confirmed by sequencing the repeat regions of several random selected individuals. Usually due to the presence of variable copy numbers of repetitive elements, the control region is considered as the source of size variation in the entire mitochondrial genome [38]. In this study, we revealed that the variable copy numbers of repetitive element could emerge in different individuals of planthoppers which were even collected from the same geographical population. This characteristic might provide additional genetic markers for studying population genetics, individual difference and phylogeographics of planthoppers.

In addition, both of these two mitochondrial genomes possess a long poly “T” stretch (23 bp) in the A+T-rich region. This poly “T” structure might be conservative in Delphacidae and be responsible for the control of replication and transcription of mitochondria.

### Base composition and AT/GC-skew of mtDNA of Hemiptera

The composition of the majority strand (J-strand) of SBPH mtDNA is A = 7099 (43.2%), T = 5581 (33.97%), G = 1527 (9.29%) and C = 2224 (13.54%). The composition of the J strand of BPH mtDNA is A = 7393 (41.96%), T = 6165 (34.99%), G = 1661 (9.43%) and C = 2400 (13.62%). Similarly to most hemipteran insects, the nucleotide compositions of the two planthopper mitogenomes are significantly biased toward A and T (BPH: 76.95%; SBPH: 77.17%). Comparing with 48 mitochondrial genomes of hemipteran species, we found that there is considerable variation in base composition among different hemipteran species: ranging from 68.9% (*Neuroctenus parus*) to 86.3% (*Aleurodicus dugesii*). These two extreme A+T content species were inferring gene arrangement [32,33]. But in different taxonomical groups of Hemiptera, the A+T% values show different ranges: Heteroptera (31 species, including 5 near complete mitochondrial genomes): 68.9-82.5%, average: 74.7±2.95%; Sternorrhyncha (9 species): 72-86.3%, average: 78.79±5.46%; Auchenorrhyncha (8 species): 75.1-78.4%, average: 76.86±0.95%. The average A+T% value for all analyzed mtDNAs set is 75.83 ± 3.67%.

The hemipteran AT-skews vary from −0.1812 (*Tria-leurodes vaporariorum*) to 0.2765 (*Lycorma delicatula*) with the BPH and SBPH mtDNA exhibiting a slight different AT-skew (0.0909 for BPH and 0.119171 for SBPH). The GC-skews of hemipteran species vary from −0.2827 (*Acyrthosiphon pisum*) to 0.2086 (*Neomaskellia andropogonis*) with the BPH and SBPH mtDNAs exhibiting very similar GC-skew (−0.1826 for BPH and −0.1842 for SBPH). Similarly, the AT/GC-skew values exhibit different ranges among three suborders: AT-skew: Heteroptera, 0.0642 - 0.2181, Sternorrhyncha, -0.1812 - 0.0956, Auchenorrhyncha, 0.0623 -T 0.2765; GC-skew: Heteroptera, -0.2656 -(−0.0965), Sternorrhyncha, -0.2810 - 0.2086, Auchenorrhyncha, -0.2827 - (−0.0783).

**Figure 6:**
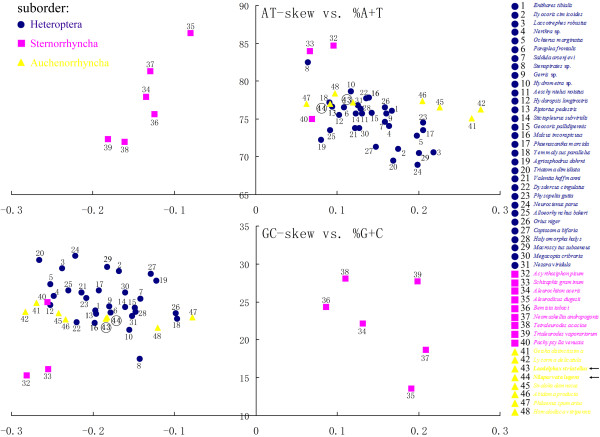
**AT% vs AT**-**skew and GC% vs GC**-**skew in the 48 hemipteran mitochondrial genomes.** Values are calculated on J-strands for full length of mt genomes. The X-axis provides the skews values, while the Y axis provides the A+T/G+C values. Names of species are colored according to their taxonomic placement (see more information of these species in Additional file 5: Table S3).

However, comparing the base compositions with the base skew values revealed that there is no correlation between the base compositions and the base skew values in any suborder of Hemiptera. For example, the A+T% values of different species of Auchenorrhyncha were much more similar than that of Heteroptera, but the range of AT/GC-skew values of this suborder was wider than Heteroptera. Even in the same suborder, the AT/GC-skew values could show great difference among different groups. Compared with other three species (belong to two families) of Sternorrhyncha, all species of Aleyrodidae exhibit converse AT/GC-skew values (Figure 6) as described in previous study [33]. These characteristics indicate that except some specific groups (species in Aleyrodidae), the base compositions and base skew values of mitochondrial genomes of hemipteran insects might be similar in low level of taxa (e.g. in the level of family) other than in the level of suborder. Furthermore, the different base compositions and base skew values to some extent reflect different taxa of Hemiptera might have suffered different evolutionary selection pressures.

The mitochondrial genome is an effective data source for resolving deep-level phylogenetic problems [39], especially in intraordinal relationships, such as in Diptera [40], Hymenoptera [41], Orthoptera [42] and Hemiptera [27]. Phylogenic analysis based on all three codon positions or first and second codon positions of PCGs can get similar results of phylogenetic relationships of Hemiptera [14,16]. But based on the rRNA genes [16] or on nuclear gene combination with mitochondrial data [15] generated a different phylogenetic relationship. However, when comparing A+T content of all PCGs, *rrnL* gene and *rrnS* gene with the entire mitochondrial genome, PCGs show more significant positive correlation (R^2^ = 0.9674) than *rrnL* gene (R^2^ = 0.8873) and *rrnS* gene (R^2^ = 0.7189) (Additional file 6: Figure S3). The PCGs seem to better reflect the evolution of the entire mitochondrial genomes than the rRNA genes. Comparing with the nuclear data set, why the mitochondrial data set result in different phylogenies in Hemiptera? Actually, many factors can influence final tree [19]: the quality of the sequences and the alignment, the amount of phylogenetic information, compositional heterogeneity, unreasonable marker choices, the accuracy of the evolutionary model and the efficiency of the tree search algorithm used. In this study, the data set of hemipteran insects show apparent base compositional heterogeneity, especially in the suborder Sternorrhyncha and Auchenorrhyncha (Figure 4). Nonstationary sequence evolution leading to unequal nucleotide composition can cause inference methods to fail and phylogenies to be inaccurate in beetles [43]. Similarly, when we chose mitochondrial data to recover the phylogenies of Hemiptera, compositional heterogeneity of mitochondrial genomes among hemipteran species should also be considered.

### Comparative analysis of two mitochondrial genomes of *L*. *striatellus*

**Figure 7:**
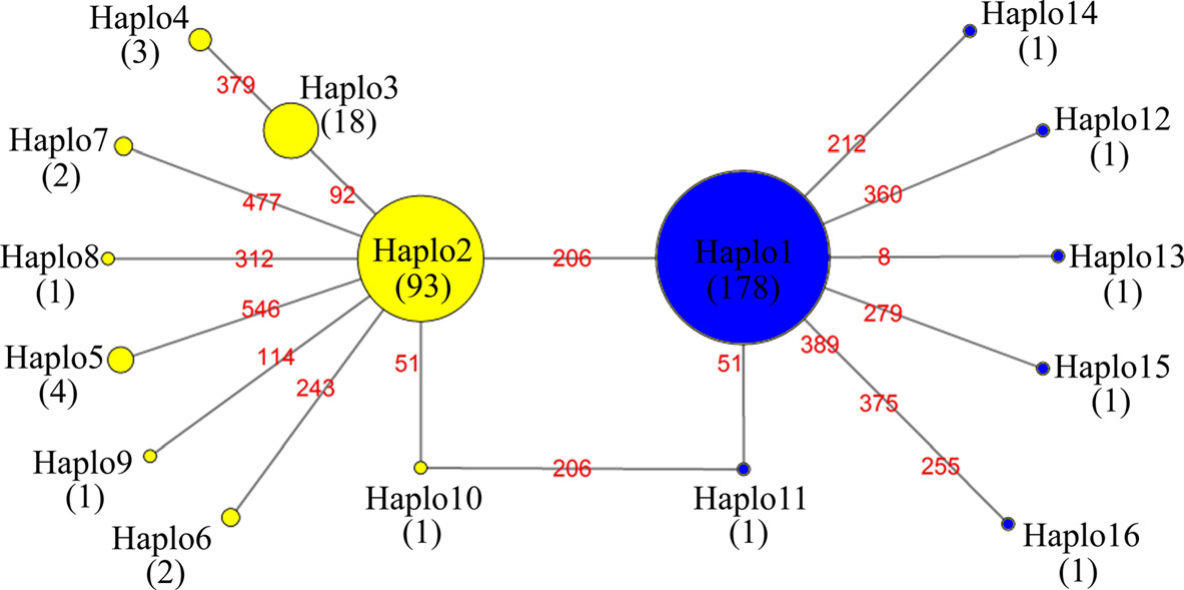
**Haplotype network based on a fragment of *Laodelphax striatellus *mitochondrial sequence (642 bp) composing of *nad5 *(482 bp), *trnH *(62 bp), 2 intergenic nucleotides and *nad4 *genes (96 bp).** Frequency of each haplotype is proportional to circle area and indicated by the numbers. The numbers on the line between circles represents the positions of each mutational site in the sequence (see more variable nucleotide site information in Additional file 8: Table S5).

The most obvious difference between the two mitochondrial genomes of *L*. *striatellus* was the location of the *trnH:* between *nad5* and *nad4* in this study just like the gene order of most hemipteran insects (Figure 3), while between *nad4L* and *nad6* in previous study [14]. Amplification of the region encompassing the *trnH* gene with the primers SBPHg4F and SBPHg4R (Additional file 7: Table S4) produced a fragment (642 bp) which is composed of portion of the *nad5* gene (482 bp), complete *trnH* gene (62 bp), two intergenic nucleotides and a portion of *nad4* gene (96 bp). All sequenced individuals (309 individuals from 15 locations) have an identical sequence of the *trnH* gene which was the same with that of the mitochondrial genome of SBPH sequenced in this study. Considering the low probability of many different sites in the *trnH* gene related sequences caused by PCR or sequencing, we still cannot exclude the possibility that some sporadic individuals of SBPH possessing the same location of *trnH* gene as previous study exist in some special regions in China. Anyway, according to the mutation sites (15 sites in *nad5* gene, one site in the intergenic region between the *trnH* gene and the *nad4* gene) of all sequences of the region compassing *trnH* gene, 16 haplotypes of the sequence were identified (Figure 7). The vast majority of individuals were belong to Haplo1 (178 individuals), Haplo2 (93 individuals) and Haplo3 (18 individuals) which were separated by two nonsynonymous nucleotide mutation at position 277 (amino acid substitution: M93L) and 391 (amino acid substitution: D131N) in *nad5* gene. There are also 4 nonsynonymous nucleotide mutations found in Haplp4, 12, 13, 14 (Additional file 8: Table S5). The cotransmission of *Wolbachia* and mitochondria causes the bacteria to have an indirect impact on the DNA diversity of the mitochondria as a result of a selective sweep of the mitotype associated with abnormal production-induced *Wolbachia* infection [44-48]. Considering the nearly 100% infection rate of *Wolbachia* and the small diversity of mitochondria (less than 0.01) were observed in natural populations (Additional file 8: Table S5), we proposed that the invasion of *Wolbachia* in SBPH occurred recently. In Jingyuan populations, all 5 individuals of SBPH were not infected with *Wolbachia* but shared mitochondrial haplotypes with other populations suggested that the *Wolbachia* might be lost in this natural population.

Another significant difference of these two mitochondrial genomes of SBPH was the distinct lengths of four NADH dehydrogenase genes (*nad2*, *nad4*, *nad5*, *nad6*) and one ATP synthase gene (*atp8*). Because of the different locations of *trnH* gene, positions of the start codon of *nad5* and *nad6* genes and the end codon of *nad4* and *nad6* genes had been changed. Comparing the region (from *nad5* to *nad6*) of mitochondrial genomes of SBPH with that of previous study, we found the differences were 57 bp deletion in the front of *nad5* gene, 21 bp deletion at the end of *nad4* gene and 84 bp insert in the front and 55 bp deletion at the end of *nad6* gene. Furthermore, there also existed 21 bp absent in the *nad2* gene and 60 bp absent at the end of *atp8* gene (Additional file 9: Table S6).

Excluding five length distinct genes (*nad2*, *nad4*, *nad5*, *nad6* and *atp8*) of mitochondrial genome of these two SBPH, the other eight PCGs and two ribosomal RNA genes represented significant difference in the evolution rates (Additional file 10: Figure S4). The *nad4L* gene which was located in the minority strand (N-strand) showed the fastest mutation rate among these genes. The mutation rates of the other PCGs ranged from 0.0022 to 0.0076. The evolution rate of *rrnS* gene (0.8%) was five times faster than *rrnL* gene (0.16%). In general, except the *trnH* and two *trnS* genes, the other tRNA genes were conserved (only few of them have single variant site).

Indeed, many insecticide resistances in arthropod pests correlated with genotype of mitochondria [2,49-52] and membrane proteins of mitochondria [53] had been reported. It is not clear whether variations found in mitochondria of planthoppers are directly implicated in resistance. Furthermore, the phenomenon that insecticide selection on heteroplasmic individuals favors those carrying resistant haplotypes in natural population had been revealed in *Tetranychus urticae*[2]. Whether similar mechanism referring to very rapid evolution and mutations being fixed occurred in planthoppers was also unknown. These aspects are well worth further study.

## Conclusion

The rearrangement of mitochondrial genome of planthoppers reported in this study adds more examples of the rearranged pattern of genes in the mitochondrial genomes of hemipteran insects studied so far. The fact that the rearrangement was conserved in Delphacidae suggests that gene rearrangements of mitochondrial genome probably occurred before the differentiation of this family. Compared with other hemipterans and other insects, the extreme short lengths of *atp8* genes found in BPH and SBPH and the additional repeats of *trnC* found in BPH raise the possibility of functional changes of these genes. The variable number of tandem repeats (VNTRs) detected in the control region might provide additional genetic markers for studying population genetics, individual difference and phylogeographics of planthoppers. The comparative analyses of two sequenced mitochondrial genomes of SBPH revealed that rearrangements of genes in the mitochondrial genome could have occurred in the same species. On account of the different locations of *trnH* gene, the start and end codon of its neighboring genes had been changed. Even the same tRNA gene (e.g. *trnS1* (AGN) and *trnS2* (UCN) genes) could form different secondary structures in these two different individuals of SBPH. All these characteristics reveal that great divergence of mitochondrial genome could occur in different individuals of a species.

## Methods

### Sample origin and DNA sequence assembly

Lab-reared populations of *Laodelphax striatellus* (Nanjing, Jiangsu province, June 2010) and *Nilaparvata lugens* (Sanya, Hainan province, August 2010) for mitochondrial genome sequence were collected in China. Other natural populations which were used to validating location of *trnH* of SBPH and detecting the variable number of tandem repeats (VNTRs) in control regions of BPH and SBPH were collected in different locations of China (Additional file 11: Table S7). Following sequencing the genome of *Wolbachia* infected in these two planthopper species, partial mtDNAs of the two planthoppers were also generated using Illumina HiSeq™ 2000. From the pair-end reads (read length: 90 bp) generated from the PCR-free random shotgun-sequencing libraries of average insert size 500 bp, one scaffold composed of three contigs of mitochondrial genome of SBPH (93,375 reads with approximately 520-fold coverage) and one scaffold composed of four contigs of mitochondrial genome of BPH (71,280 reads with approximately 400-fold coverage) were generated (Figure 1).

### Gap closing-PCR amplification, cloning and sequencing

According to the flanking sequences of the gaps assembled from the filtered data, we designed 11 pairs of perfectly matched primers to connect the adjacent contigs (Additional file 7: Table S4). In order to reduce the error probability, we chose the high fidelity DNA polymerase Phusion® High-Fidelity DNA Polymerase (New England Biolabs) to perform the PCR amplification. The cycling conditions: 98°C 30 seconds, followed by 35 cycles of 10 s at 98°C, 30 s at different annealing temperatures, 1–2.5 min at 72°C depending on the size of amplicons, and the subsequent final elongation step at 72°C for 10 mins. The quality of PCR products was examined by 1% agarose gel electrophoresis. After purification, PCR products which were shorter than 800 bp were directly sequenced from both directions using the ABI 3730XL Genetic Analyzer (PE Applied Biosystems). Otherwise, the PCR fragments (>800 bp) were ligated into pEASY-Blunt cloning vector (Beijing TransGen Biotech) and resulting plasmid DNAs were isolated and used for sequencing. For each larger PCR product, at least two independent clones were sequenced to ensure that we obtained the consistent sequence. The flanking sequences of each gap were exactly matched with the sequences generated based on PCR method. For closing the gaps which contain the AT-rich region and detecting the variable number of tandem repeats (VNTRs) in natural population individuals, we chose the PrimeSTAR GXL DNA polymerase (TAKARA) to perform the PCR amplification. The complete sequences of mitochondrial genome of SBPH and BPH are deposited in the GenBank database under the Accession No. JX880068, JX880069 respectively.

### Annotation and analysis

After finished the gap closing work, we annotated the mitochondrial genomes of BPH and SBPH. All PCGs were identified by ORF Finder implemented at the NCBI website with the invertebrate mitochondrial genetic codes. Finally pair-wise comparisons with orthologous proteins from other hemipteran insects were performed to better define the limits of PCGs. The transfer RNA (tRNA) genes were identified using both of the tRNAscan-SE [54] and ARWEN [55] programs and recognized manually as sequences having the appropriate anticodon and capable of folding into the typical cloverleaf secondary structure. The locations of the two rRNA genes (*rrnL* and *rrnS*) were determined based on alignments and secondary structures of rRNA sequences of other hemipteran species. The boundaries of the ribosomal *rrnL* gene were assumed to be delimited by the ends of the *trnL1* (CUN) and *trnV*. The 3′ end of *rrnS* gene was assumed to be delimited by the start of *trnV* while the 5′ end was determined through comparison with orthologous genes of other hemipteran species sequenced so far. The base composition, the Relative Synonymous Codon Usage (RSCU) values of BPH and SBPH were calculated with MEGA 4 program [56].

### Comparative genomics analysis

After downloaded other mitochondrioal genome sequence (Heteroptera, 31 species; Sternorrhyncha, 9 species; Auchenorrhyncha, 7 species, Additional file 5: Table S3), we compared the two planthopper species with other hemipteran species, including the gene order, the base composition and the GC/AT-skew. The GC-skew = (G-C)/(G+C) and AT-skew = (A-T)/(A+T) were used [57] to measure the base compositional difference between different taxa in Hemiptera. In addition, in order to reveal whether two *trnH* rearrangement types really exist in natural populations, we sequenced a region of mitochondrial genome encompassing the *trnH* gene from 309 individuals of SBPH which were collected from 15 natural populations and also checked the *Wolbachia* infection status of each individual (Additional file 11: Table S7). After got the sequences, we used Network 4.610 (http://www.fluxus-engineering.com) to calculate the frequency of each haplotype of the sequence of this region. The sequences of each haplotype are deposited in the GenBank database under the Accession No. KC006945 - KC006960.

## Competing interests

The authors declare that they have no competing interests.

## Authors’ contributions

KZ conceived and designed the experiments, carried out the data analyses and drafted the manuscript. XH directed and coordinated this study and revised the manuscript. KZ, WZ, XR and YZ carried out molecular experiments. WZ, XD, JL and DC collected the samples and helped with DNA extraction. YD helped with the sequence reads assembly. All authors read and approved the final manuscript.

## Supplementary Material

Additional file 1: Table S1 Annotation for the mitochondrial genome of *Nilaparvata lugens*. Click here for file

Additional file 2: Figure S1 Putative secondary structures of the 22 tRNA genes identified in the mitochondrial genome of *Nilaparvata lugens*. All tRNA genes are shown in the order of occurrence in the mitochondrial genome starting from *trnI*. Bars indicate Watson-Crick base pairings, and dots between G and U pairs mark canonical base pairings appearing in tRNA. Click here for file

Additional file 3: Figure S2 Putative secondary structures of the 22 tRNA genes identified in the mitochondrial genome of *Laodelphax striatellus*. All tRNA genes are shown in the order of occurrence in the mitochondrial genome starting from *trnI*. Bars indicate Watson-Crick base pairings, and dots between G and U pairs mark canonical base pairings appearing in tRNA. Click here for file

Additional file 4: Table S2 Annotation for the mitochondrial genome of *Laodelphax striatellus*. Click here for file

Additional file 5: Table S3 Information concerning the hemipteran species with complete or nearly complete mitochondrial genome used in this study. Click here for file

Additional file 6: Figure S3 Row correlation of the A+T content values between the 13 PCGs, *rrnL* gene and *rrnS* gene and the entire mitochondrial genome in all hemipteran insects sequenced so far. Click here for file

Additional file 7: Table S4 The primers used for the PCR analysis and gap closing of two mitochondrial genomes in the present study. Click here for file

Additional file 8: Table S5 Variable nucleotide site information of the “*nad5*-*trnH*-*nad4*” region in the mitochondrial genome of *Laodelphax striatellus*. Click here for file

Additional file 9: Table S6 Comparison and re-annotation of previously studied mitochondrial genomes of *Laodelphax striatellus*. Click here for file

Additional file 10: Figure S4 The mutation rates of each gene between mitochondrial genomes of two individuals of *Laodelphax striatellus*. The other PCGs (*nad2*, *atp8*, *nad5*, *nad4* and *nad6*) were not calculated for the variant length of these genes in these two individual mitochondria. Click here for file

Additional file 11: Table S7 Collection information for natural populations of *Nilaparvata lugens* and *Laodelphax striatellus* used in this study. Click here for file
