# Supplementary material for: The complete mitochondrial genomes of two rice planthoppers, Nilaparvata lugens and Laodelphax striatellus: conserved genome rearrangement in Delphacidae and discovery of new characteristics of atp8 and tRNA genes
Source: BMC Genomics. 2013 Jun 22;14:417. doi: 10.1186/1471-2164-14-417 (PMC3701526; doi:10.1186/1471-2164-14-417)
Supplement: Additional file 1: Table S1 — Annotation for the mitochondrial genome of Nilaparvata lugens. [file 1471-2164-14-417-S1.doc]

Table S1. Annotation for the mitogenome of *Nilaparvata lugens*.

| Gene | Direction | Start | End | Size | Intergenic nucleotides | First codon | Stop codon | Anticodon |
| --- | --- | --- | --- | --- | --- | --- | --- | --- |
| *trnI* | F | 1 | 69 | 69 |  |  |  | GAT(30-32) |
| *trnQ* | R | 73 | 139 | 67 | 3 |  |  | TTG(110-108) |
| *trnM* | F | 139 | 202 | 64 | -1 |  |  | CAT(171-173) |
| *nad2* | F | 203 | 1156 | 954 | 0 | ATT | TAA |  |
| *trnC* | R | 1157 | 1219 | 63 | 0 |  |  | GCA(1188-1190) |
| *trnC* | R | 1568 | 1630 | 63 | 348 |  |  | GCA(1599-1601) |
| *trnC* | R | 1979 | 2041 | 63 | 348 |  |  | GCA(2010-2012) |
| *trnW* | F | 2052 | 2116 | 65 | 10 |  |  | TCA(2082-2084) |
| *trnY* | R | 2126 | 2188 | 63 | 9 |  |  | GTA(2157-2159) |
| *cox1* | F | 2202 | 3735 | 1534 | 13 | ATG | T |  |
| *trnL2* (UUR) | F | 3736 | 3801 | 66 | 0 |  |  | TAA(3765-3767) |
| *cox2* | F | 3802 | 4464 | 663 | 0 | ATT | TAA |  |
| *trnK* | F | 4476 | 4546 | 71 | 11 |  |  | CTT(4506-4508) |
| *trnD* | F | 4547 | 4610 | 64 | 0 |  |  | GTC(4578-4580) |
| *atp8* | F | 4611 | 4709 | 99 | 0 | ATT | TAA |  |
| *atp6* | F | 4709 | 5360 | 652 | -1 | ATA | T |  |
| *cox3* | F | 5361 | 6138 | 778 | 0 | ATG | T |  |
| *trnG* | F | 6139 | 6200 | 62 | 0 |  |  | TCC(6169-6171) |
| *nad3* | F | 6201 | 6551 | 351 | 0 | ATA | TAA |  |
| *trnA* | F | 6553 | 6613 | 61 | 1 |  |  | TGC(6582-6584) |
| *trnR* | F | 6618 | 6677 | 60 | 4 |  |  | TCG(6645-6647) |
| *trnN* | F | 6677 | 6740 | 64 | -1 |  |  | GTT(6707-6709) |
| *trnS1* (AGN) | F | 6741 | 6797 | 57 | 0 |  |  | GCT(6761-6763) |
| *trnE* | F | 6798 | 6862 | 65 | 0 |  |  | TTC(6829-6831) |
| *trnF* | R | 6863 | 6928 | 66 | 0 |  |  | GAA(6896-6894) |
| *nad5* | R | 6929 | 8603 | 1675 | 0 | ATG | T |  |
| *trnH* | R | 8604 | 8664 | 61 | 0 |  |  | GTG(8631-8633) |
| *nad4* | R | 8664 | 9980 | 1317 | -1 | ATG | TAA |  |
| *nad4l* | R | 9974 | 10243 | 270 | -7 | ATG | TAA |  |
| *nad6* | F | 10277 | 10816 | 540 | 33 | ATT | TAA |  |
| *trnP* | R | 10865 | 10926 | 62 | 48 |  |  | TGG(10894-10896) |
| *trnT* | F | 10927 | 10990 | 64 | 0 |  |  | TGT(10959-10961) |
| *cytb* | F | 10998 | 12101 | 1104 | 7 | ATG | TAG |  |
| *trnS2* (UCN) | F | 12092 | 12153 | 62 | -10 |  |  | TGA(12121-12123) |
| *nad1* | R | 12174 | 13091 | 918 | 20 | ATG | TAG |  |
| *trnL1* (CUN) | R | 13093 | 13154 | 62 | 1 |  |  | TAG(13125-13123) |
| *rrnL* | R | 13155 | 14373 | 1219 | 0 |  |  |  |
| *trnV* | R | 14373 | 14443 | 71 | -1 |  |  | TAC(14412-14414) |
| *rrnS* | R | 14443 | 15190 | 748 | -1 |  |  |  |
| AT-rich |  | 15191 | 17619 | 2429 |  |  |  |  |
| repeat region |  | 16245 | 17398 | 1154 |  |  |  |  |
