# Supplementary figures and images for: The complete mitochondrial genomes of two rice planthoppers, Nilaparvata lugens and Laodelphax striatellus: conserved genome rearrangement in Delphacidae and discovery of new characteristics of atp8 and tRNA genes
Source: BMC Genomics. 2013 Jun 22;14:417. doi: 10.1186/1471-2164-14-417 (PMC3701526; doi:10.1186/1471-2164-14-417)

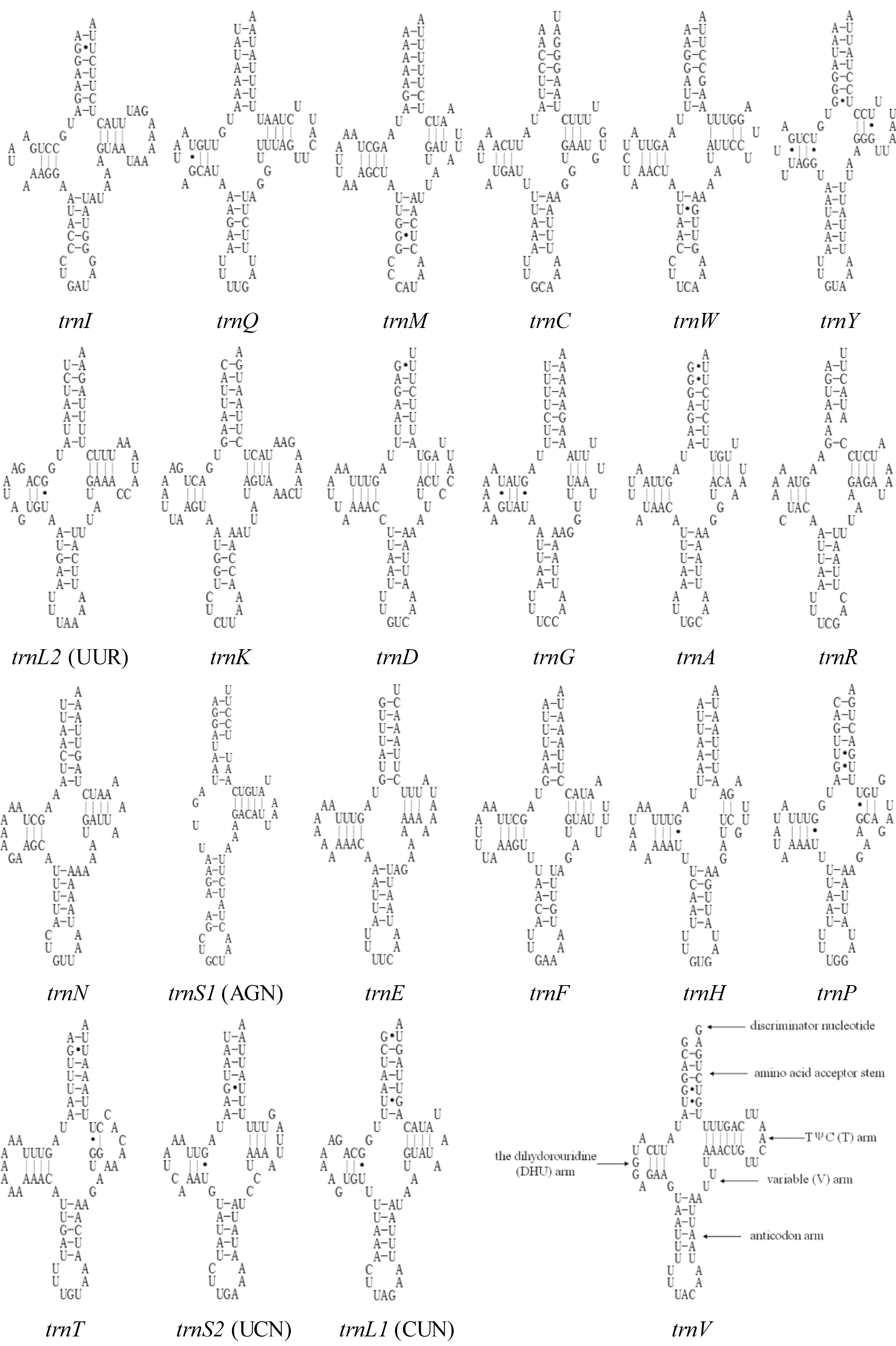

Supplement: Additional file 2: Figure S1 — Putative secondary structures of the 22 tRNA genes identified in the mitochondrial genome of Nilaparvata lugens. All tRNA genes are shown in the order of occurrence in the mitochondrial genome starting from trnI. Bars indicate Watson-Crick base pairings, and dots between G and U pairs mark canonical base pairings appearing in tRNA. [file 1471-2164-14-417-S2.tiff]

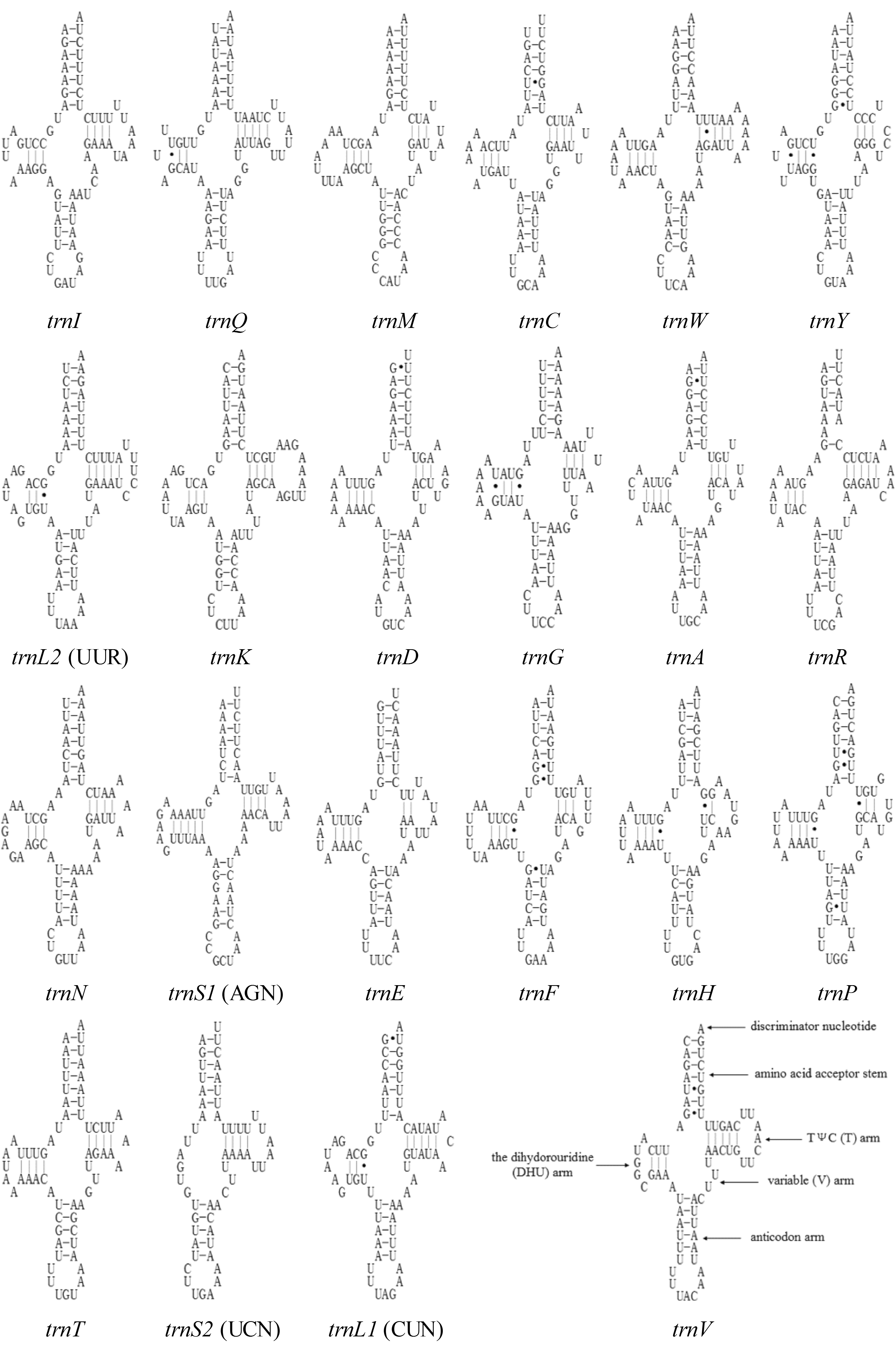

Supplement: Additional file 3: Figure S2 — Putative secondary structures of the 22 tRNA genes identified in the mitochondrial genome of Laodelphax striatellus. All tRNA genes are shown in the order of occurrence in the mitochondrial genome starting from trnI. Bars indicate Watson-Crick base pairings, and dots between G and U pairs mark canonical base pairings appearing in tRNA. [file 1471-2164-14-417-S3.tiff]

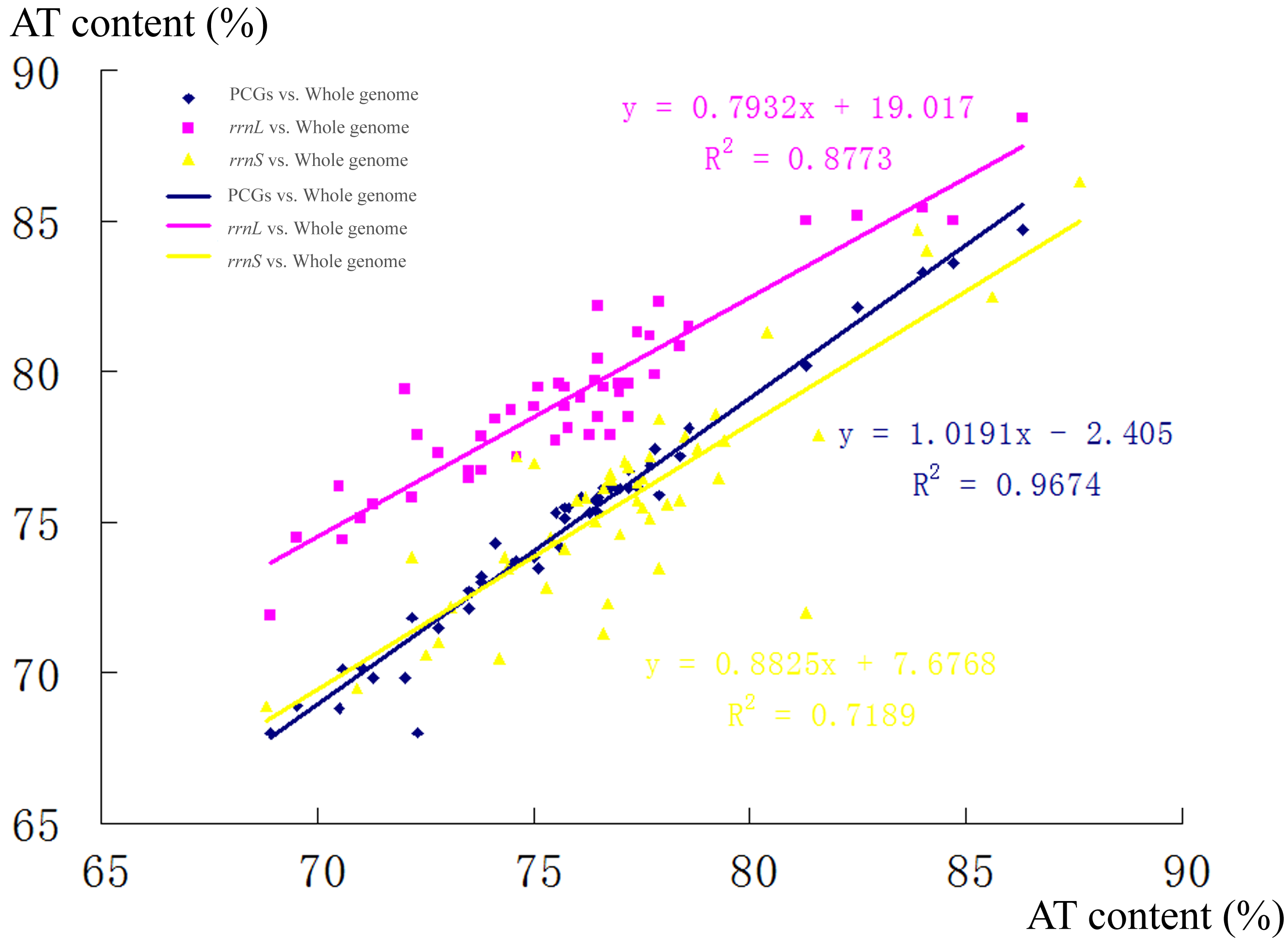

Supplement: Additional file 6: Figure S3 — Row correlation of the A+T content values between the 13 PCGs, rrnL gene and rrnS gene and the entire mitochondrial genome in all hemipteran insects sequenced so far. [file 1471-2164-14-417-S6.tiff]

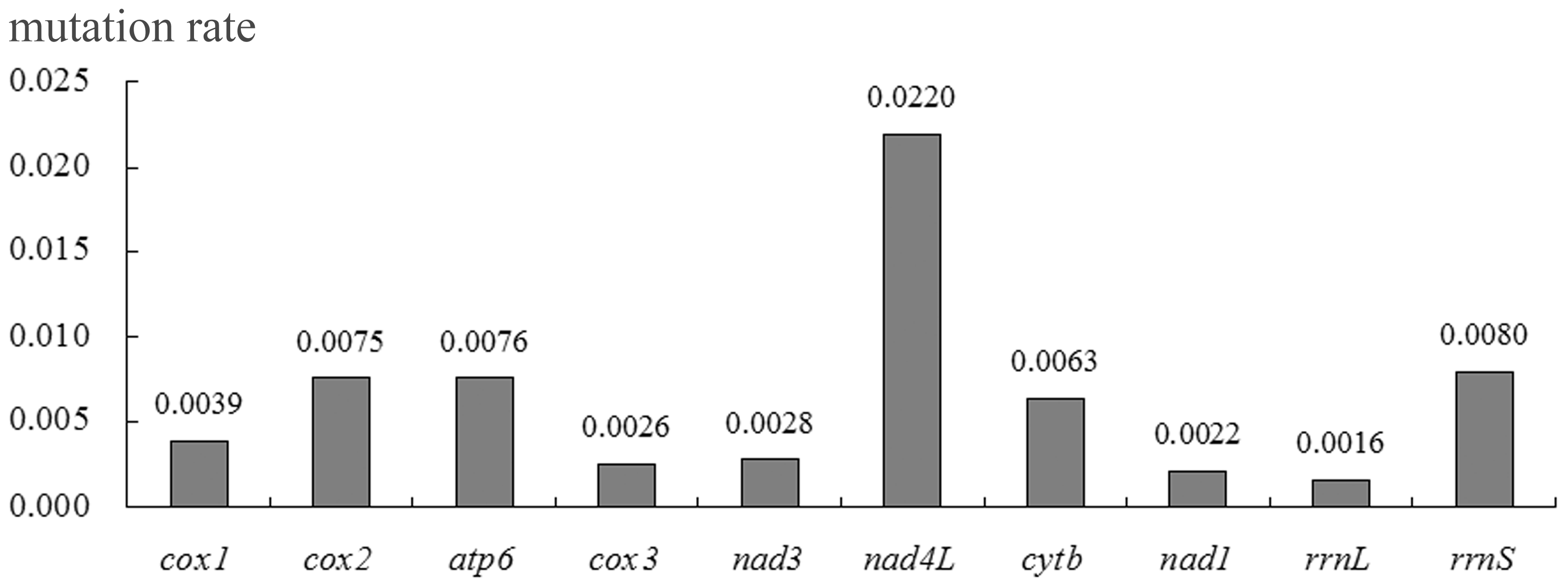

Supplement: Additional file 10: Figure S4 — The mutation rates of each gene between mitochondrial genomes of two individuals of Laodelphax striatellus. The other PCGs (nad2, atp8, nad5, nad4 and nad6) were not calculated for the variant length of these genes in these two individual mitochondria. [file 1471-2164-14-417-S10.tiff]
