# Supplementary material for: The complete mitochondrial genomes of two rice planthoppers, Nilaparvata lugens and Laodelphax striatellus: conserved genome rearrangement in Delphacidae and discovery of new characteristics of atp8 and tRNA genes
Source: BMC Genomics. 2013 Jun 22;14:417. doi: 10.1186/1471-2164-14-417 (PMC3701526; doi:10.1186/1471-2164-14-417)
Supplement: Additional file 4: Table S2 — Annotation for the mitochondrial genome of Laodelphax striatellus. [file 1471-2164-14-417-S4.doc]

Table S2. Annotation for the mitogenome of *Laodelphax striatellus*.

| Gene | Direction | Start | End | Size | Intergenic nucleotides | First codon | Stop codon | Anticodon |
| --- | --- | --- | --- | --- | --- | --- | --- | --- |
| *trnI* | F | 1 | 66 | 66 |  |  |  | GAT(30–32) |
| *trnQ* | R | 69 | 134 | 66 | 2 |  |  | TTG(106-104) |
| *trnM* | F | 134 | 198 | 65 | -1 |  |  | CAT(167–169) |
| *nad2* | F | 199 | 1155 | 957 | 0 | ATT | TAA |  |
| *trnC* | R | 1159 | 1220 | 62 | 3 |  |  | GCA(1191-1189) |
| *trnW* | F | 1255 | 1321 | 67 | 34 |  |  | TCA(1285-1287) |
| *trnY* | R | 1325 | 1385 | 61 | 3 |  |  | GTA(1354-1356) |
| *cox1* | F | 1390 | 2923 | 1534 | 4 | ATG | T |  |
| *trnL2* (UUR) | F | 2924 | 2989 | 66 | 0 |  |  | TAA(2953-2955) |
| *cox2* | F | 2990 | 3652 | 663 | 0 | ATT | TAG |  |
| *trnK* | F | 3653 | 3723 | 71 | 0 |  |  | CTT(3683-3685) |
| *trnD* | F | 3724 | 3785 | 62 | 0 |  |  | GTC(3754-3756) |
| *atp8* | F | 3786 | 3887 | 102 | 0 | ATT | TAA |  |
| *atp6* | F | 3881 | 4535 | 655 | -7 | ATG | T |  |
| *cox3* | F | 4536 | 5316 | 781 | 0 | ATG | T |  |
| *trnG* | F | 5317 | 5377 | 61 | 0 |  |  | TCC(5347-5349) |
| *nad3* | F | 5378 | 5728 | 351 | 0 | ATT | TAA |  |
| *trnA* | F | 5728 | 5788 | 61 | -1 |  |  | TGC(5757-5759) |
| *trnR* | F | 5793 | 5855 | 63 | 4 |  |  | TCG(5821-5823) |
| *trnN* | F | 5855 | 5918 | 64 | -1 |  |  | GTT(5885-5887) |
| *trnS1* (AGN) | F | 5905 | 5973 | 69 | -14 |  |  | GCT(5938-5940) |
| *trnE* | F | 5974 | 6035 | 62 | 0 |  |  | TTC(6004-6006) |
| *trnF* | R | 6035 | 6099 | 65 | -1 |  |  | GAA(6067-6065) |
| *nad5* | R | 6100 | 7774 | 1675 | 0 | ATG | T |  |
| *trnH* | R | 7775 | 7836 | 62 | 0 |  |  | GTG(7804-7806) |
| *nad4* | R | 7839 | 9155 | 1317 | 2 | ATG | TAA |  |
| *nad4l* | R | 9149 | 9421 | 273 | -7 | ATG | TAA |  |
| *nad6* | F | 9467 | 10006 | 540 | 45 | ATT | TAA |  |
| *trnP* | R | 10065 | 10126 | 62 | 58 |  |  | TGG(10094-10096) |
| *trnT* | F | 10131 | 10193 | 63 | 4 |  |  | TGT(10161-10163) |
| *cytb* | F | 10198 | 11301 | 1104 | 4 | ATG | TAG |  |
| *trnS2* (UCN) | F | 11300 | 11355 | 56 | -2 |  |  | TGA(11320-12322) |
| *nad1* | R | 11373 | 12290 | 918 | 17 | ATG | TAG |  |
| *trnL1* (CUN) | R | 12292 | 12355 | 64 | 1 |  |  | TAG(12326-12324) |
| *rrnL* | R | 12356 | 13574 | 1219 | 0 |  |  |  |
| *trnV* | R | 13575 | 13642 | 68 | 0 |  |  | TAC(13612-13614) |
| *rrnS* | R | 13643 | 14389 | 747 | 0 |  |  |  |
| AT-rich |  | 14390 | 16431 | 2042 | 0 |  |  |  |
| repeat region |  | 15488 | 16216 | 729 |  |  |  |  |
