# Supplementary material for: The complete mitochondrial genomes of two rice planthoppers, Nilaparvata lugens and Laodelphax striatellus: conserved genome rearrangement in Delphacidae and discovery of new characteristics of atp8 and tRNA genes
Source: BMC Genomics. 2013 Jun 22;14:417. doi: 10.1186/1471-2164-14-417 (PMC3701526; doi:10.1186/1471-2164-14-417)
Supplement: Additional file 5: Table S3 — Information concerning the hemipteran species with complete or nearly complete mitochondrial genome used in this study. [file 1471-2164-14-417-S5.doc]

Table S3. Information concerning the hemipteran species with complete or nearly complete mitochondrial genome used in this study.

| ACCN | Species | Suborder | Superfamily | Family | Length |
| --- | --- | --- | --- | --- | --- |
| NC_012819 | *Enithares tibialis* | Heteroptera | - | Notonectidae | 15262 |
| NC_012845 | *Ilyocoris cimicoides* | Heteroptera | - | Naucoridae | 15209 |
| NC_012817 | *Laccotrephes robustus* | Heteroptera | - | Nepidae | 15321 |
| NC_012838 | *Nerthra sp. NKMT022* | Heteroptera | - | Gelastocoridae | 16079 |
| NC_012820 | *Ochterus marginatus* | Heteroptera | - | Ochteridae | 14609 |
| NC_012822 | *Paraplea frontalis* | Heteroptera | - | Pleidae | 15130 |
| NC_012463 | *Saldula arsenjevi* | Heteroptera | - | Saldidae | 15324 |
| NC_016017 | *Stenopirates sp. HL-2011* | Heteroptera | - | Enicocephalidae | 15384 |
| NC_012841 | *Gerris sp. NKMT033* | Heteroptera | Gerroidea | Gerridae | 15380 |
| NC_012842 | *Hydrometra sp. NKMT020* | Heteroptera | Gerroidea | Hydrometridae | 15416 |
| NC_012446 | *Aeschyntelus notatus* | Heteroptera | Coreoidea | Rhopalidae | 14532* |
| NC_012456 | *Hydaropsis longirostris* | Heteroptera | Coreoidea | Coreidae | 16521 |
| NC_012462 | *Riptortus pedestris* | Heteroptera | Coreoidea | Alydidae | 17191 |
| NC_012888 | *Stictopleurus subviridis* | Heteroptera | Coreoidea | Rhopalidae | 15319 |
| NC_012424 | *Geocoris pallidipennis* | Heteroptera | Lygaeoidea | Lygaeidae | 14592* |
| NC_012458 | *Malcus inconspicuus* | Heteroptera | Lygaeoidea | Malcidae | 15575 |
| NC_012460 | *Phaenacantha marcida* | Heteroptera | Lygaeoidea | Colobathristidae | 14540* |
| NC_012464 | *Yemmalysus parallelus* | Heteroptera | Lygaeoidea | Berytidae | 15747 |
| NC_015842 | *Agriosphodrus dohrni* | Heteroptera | Reduvioidea | Reduviidae | 16470 |
| NC_002609 | *Triatoma dimidiata* | Heteroptera | Reduvioidea | Reduviidae | 17019 |
| NC_012823 | *Valentia hoffmanni* | Heteroptera | Reduvioidea | Reduviidae | 15625 |
| NC_012421 | *Dysdercus cingulatus* | Heteroptera | Pyrrhocoroidea | Pyrrhocoridae | 16249 |
| NC_012432 | *Physopelta gutta* | Heteroptera | Pyrrhocoroidea | Largidae | 14935 |
| NC_012459 | *Neuroctenus parus* | Heteroptera | Aradoidea | Aradidae | 15354 |
| NC_016432 | *Alloeorhynchus bakeri* | Heteroptera | Cimicoidea | Nabidae | 15851 |
| NC_012429 | *Orius niger* | Heteroptera | Cimicoidea | Anthocoridae | 14494* |
| NC_012449 | *Coptosoma bifaria* | Heteroptera | Pentatomoidea | Plataspidae | 16179 |
| NC_013272 | *Halyomorpha halys* | Heteroptera | Pentatomoidea | Pentatomidae | 16518 |
| NC_012457 | *Macroscytus subaeneus* | Heteroptera | Pentatomoidea | Cydnidae | 14620* |
| NC_015342 | *Megacopta cribraria* | Heteroptera | Pentatomoidea | Plataspidae | 15647 |
| NC_011755 | *Nezara viridula* | Heteroptera | Pentatomoidea | Pentatomidae | 16889 |
| NC_011594 | *Acyrthosiphon pisum* | Sternorrhyncha | Aphidoidea | Aphididae | 16971 |
| NC_006158 | *Schizaphis graminum* | Sternorrhyncha | Aphidoidea | Aphididae | 15721 |
| NC_006160 | *Aleurochiton aceris* | Sternorrhyncha | Aleyrodoidea | Aleyrodidae | 15388 |
| NC_005939 | *Aleurodicus dugesii* | Sternorrhyncha | Aleyrodoidea | Aleyrodidae | 15723 |
| NC_006279 | *Bemisia tabaci* | Sternorrhyncha | Aleyrodoidea | Aleyrodidae | 15322 |
| NC_006159 | *Neomaskellia andropogonis* | Sternorrhyncha | Aleyrodoidea | Aleyrodidae | 14496 |
| NC_006292 | *Tetraleurodes acaciae* | Sternorrhyncha | Aleyrodoidea | Aleyrodidae | 15080 |
| NC_006280 | *Trialeurodes vaporariorum* | Sternorrhyncha | Aleyrodoidea | Aleyrodidae | 18414 |
| NC_006157 | *Pachypsylla venusta* | Sternorrhyncha | Psylloidea | Psyllidae | 14711 |
| NC_012617 | *Geisha distinctissima* | Auchenorrhyncha | Fulgoroidea | Flatidae | 15971 |
| NC_013706 | *Laodelphax striatellus* | Auchenorrhyncha | Fulgoroidea | Delphacidae | 16513 |
| NC_012835 | *Lycorma delicatula* | Auchenorrhyncha | Fulgoroidea | Fulgoridae | 15410 |
| NC_014286 | *Sivaloka damnosus* | Auchenorrhyncha | Fulgoroidea | Issidae | 15287 |
| NC_015799 | *Abidama producta* | Auchenorrhyncha | Cercopoidea | Cercopidae | 15277 |
| NC_005944 | *Philaenus spumarius* | Auchenorrhyncha | Cercopoidea | Aphrophoridae | 16324 |
| NC_006899 | *Homalodisca vitripennis* | Auchenorrhyncha | Membracoidea | Cicadellidae | 15304 |

"-" means the supfarmily of the species was undetermined. The species marked with red color mean existing gene arrangements in mitochondrial genome. “*” means the mitochondrial genome sequence was nearly complete.
