# Supplementary material for: The complete mitochondrial genomes of two rice planthoppers, Nilaparvata lugens and Laodelphax striatellus: conserved genome rearrangement in Delphacidae and discovery of new characteristics of atp8 and tRNA genes
Source: BMC Genomics. 2013 Jun 22;14:417. doi: 10.1186/1471-2164-14-417 (PMC3701526; doi:10.1186/1471-2164-14-417)
Supplement: Additional file 7: Table S4 — The primers used for the PCR analysis and gap closing of two mitochondrial genomes in the present study. [file 1471-2164-14-417-S7.doc]

Table S4. The primers used for the PCR analysis and gap closing of two mitochondrial genomes in the present study.

| Species | Primer (position in mitochondrial genome) | Sequence (5'-3') | Annealing Temperature |
| --- | --- | --- | --- |
| *Laodelphax striatellus* | SBPHg1F (14247-14265) | ACTAAAGTTACCCCCCAAT | 55°C |
|  | SBPHg1R (16381-16399) | TTTACACCTCTTTTTCCCA |  |
|  |  |  |  |
|  | SBPHg2F (3491-3509) | ATAGATGCCGTACCAGGAC | 55°C |
|  | SBPHg2R (4267-4284) | ATTGGAGTTGGTGTGTTT |  |
|  |  |  |  |
|  | SBPHg3F (6162-6183) | AAATGTAAAGAATCCAAGTGTA | 55°C |
|  | SBPHg3R (6724-6742) | TTATGTGGTGTACCATTTT |  |
|  |  |  |  |
|  | SBPHg4F (7272-7292) | TAGAAATAATGTCAACAACCA | 55°C |
|  | SBPHg4R (7935-7954) | ATACCTTTGGTCTTGTTCTT |  |
|  |  |  |  |
|  | SBPHg5F (8952-8970) | AAATAACCAAAAAGTCAAC | 55°C |
|  | SBPHg5R (10238-10257) | ATTAGAAGGGGTAGGCAGGT |  |
|  |  |  |  |
|  | SBPHg6F (9204-9221) | ACTTAAAAGGGTCAAAGAAAT | 55°C |
|  | SBPHg6R (10116-10133) | TTAGAAGGGGTAGGCAGGT |  |
|  |  |  |  |
| *Nilaparvata lugens* | BPHg1F (649-667) | TATCGGAAGATTAAGAGGA | 54°C |
|  | BPHg1R (2262-2279) | AAATCCTGACCAAATACC |  |
|  |  |  |  |
|  | BPHg2F (8387-8405) | ATAAAAAGACACACCCAGA | 51°C |
|  | BPHg2R (9292-9310) | ATGGTCTTATTCGTGTTCT |  |
|  |  |  |  |
|  | BPHg3F (10560-10578) | TAACCTCAACAGTCATAAA | 51°C |
|  | BPHg3R (11284-11302) | AGGAAAGAGCCATAGTAAA |  |
|  |  |  |  |
|  | BPHg4F (15437-15455) | AATAAATCCTTCCACTAAA | 46°C |
|  | BPHg4R (16205-16223) | TCAAAAAAAATGCACCAAA |  |
|  |  |  |  |
|  | BPHg5F (16042-16064) | AATTTTTTAATCTCTTATTTCGT | 51°C |
|  | BPHg5R (278-300) | ATAATTCATATTGAAAATCAGTT |  |
