# Supplementary material for: The complete mitochondrial genomes of two rice planthoppers, Nilaparvata lugens and Laodelphax striatellus: conserved genome rearrangement in Delphacidae and discovery of new characteristics of atp8 and tRNA genes
Source: BMC Genomics. 2013 Jun 22;14:417. doi: 10.1186/1471-2164-14-417 (PMC3701526; doi:10.1186/1471-2164-14-417)
Supplement: Additional file 8: Table S5 — Variable nucleotide site information of the “nad5-trnH-nad4” region in the mitochondrial genome of Laodelphax striatellus. [file 1471-2164-14-417-S8.doc]

Table S5. Variable nucleotide site information of the "*nad5*-*trnH*-*nad4*" region in the mitochondrial genome of *Laodelphax striatellus*.

| Haplotype (GenBank accession number) | Nucleotide position* | | | | | | | | | | | | | | | |
| --- | --- | --- | --- | --- | --- | --- | --- | --- | --- | --- | --- | --- | --- | --- | --- | --- |
|  | 8 | 51 | 92 | 114 | 206 | 212 | 243 | 255 | 279 | 312 | 360 | 375 | 379 | 389 | 477 | 546 |
|  | 7300 | 7343 | 7384 | 7406 | 7498 | 7504 | 7535 | 7547 | 7571 | 7604 | 7652 | 7667 | 7671 | 7681 | 7769 | 7838 |
|  | 475 | 432 | 391 | 369 | 277 | 271 | 240 | 228 | 204 | 171 | 123 | 108 | 104 | 94 | 6 | - |
|  | 1 | 3 | 1 | 3 | 1 | 1 | 3 | 3 | 3 | 3 | 3 | 3 | 2 | 1 | 3 | - |
| Haplo1 (KC006945) | T | G | C | A | T | C | A | G | G | C | T | A | G | A | C | C |
| Haplo2 (KC006946) | · | · | · | · | **A** | · | · | · | · | · | · | · | · | · | · | · |
| Haplo3 (KC006947) | · | · | **T** | · | **A** | · | · | · | · | · | · | · | · | · | · | · |
| Haplo4 (KC006948) | · | · | **T** | · | **A** | · | · | · | · | · | · | · | **C** | · | · | · |
| Haplo5 (KC006949) | · | · | · | · | **A** | · | · | · | · | · | · | · | · | · | · | T |
| Haplo6 (KC006950) | · | · | · | · | **A** | · | G | · | · | · | · | · | · | · | · | · |
| Haplo7 (KC006951) | · | · | · | · | **A** | · | · | · | · | · | · | · | · | · | T | · |
| Haplo8 (KC006952) | · | · | · | · | **A** | · | · | · | · | T | · | · | · | · | · | · |
| Haplo9 (KC006953) | · | · | · | G | **A** | · | · | · | · | · | · | · | · | · | · | · |
| Haplo10 (KC006954) | · | A | · | · | **A** | · | · | · | · | · | · | · | · | · | · | · |
| Haplo11 (KC006955) | · | A | · | · | · | · | · | · | · | · | · | · | · | · | · | · |
| Haplo12 (KC006956) | · | · | · | · | · | · | · | · | · | · | **A** | · | · | · | · | · |
| Haplo13 (KC006957) | **C** | · | · | · | · | · | · | · | · | · | · | · | · | · | · | · |
| Haplo14 (KC006958) | · | · | · | · | · | **T** | · | · | · | · | · | · | · | · | · | · |
| Haplo15 (KC006959) | · | · | · | · | · | · | · | · | A | · | · | · | · | · | · | · |
| Haplo16 (KC006960) | · | · | · | · | · | · | · | A | · | · | · | G | · | G | · | · |

*: means extensional positions of each site: first line, in the alignment; second line, in the mitochondrial genome; third line, in nad5 gene; fourth line, in codon position. "·" means the nucleotide of this site was the same with Haplo1. "-" means the site was located in the intergenic region of *trnH* and *nad4* genes. The nonsynonymous mutation sites were marked with red color.
